# An Outbreak of Carbapenem-Resistant and Hypervirulent *Klebsiella pneumoniae* in an Intensive Care Unit of a Major Teaching Hospital in Wenzhou, China

**DOI:** 10.3389/fpubh.2019.00229

**Published:** 2019-08-19

**Authors:** Yajie Zhao, Xiucai Zhang, Von Vergel L. Torres, Haiyang Liu, Andrea Rocker, Yizhi Zhang, Jiawei Wang, Lijiang Chen, Wenzi Bi, Jie Lin, Richard A. Strugnell, Siqin Zhang, Trevor Lithgow, Tieli Zhou, Jianming Cao

**Affiliations:** ^1^School of Laboratory Medicine and Life Science, Wenzhou Medical University, Wenzhou, China; ^2^Department of Clinical Laboratory, The First Affiliated Hospital of Wenzhou Medical University, Wenzhou, China; ^3^Infection and Immunity Program, Department of Microbiology, Biomedicine Discovery Institute, Monash University, Melbourne, VIC, Australia; ^4^School of Medicine, The Fourth Affiliated Hospital of Zhejiang University, Jinhua, China; ^5^Department of Microbiology and Immunology, The Peter Doherty Institute, The University of Melbourne, Melbourne, VIC, Australia

**Keywords:** *Klebsiella pneumoniae*, hypervirulent, carbapenem-resistance, ST11, epidemiology

## Abstract

Carbapenem-resistant, hypervirulent *Klebsiella pneumoniae* (CR-hvKP) has recently emerged as a significant threat to public health. In this study, 29 *K. pneumoniae* isolates were isolated from eight patients admitted to the intensive care unit (ICU) of a comprehensive teaching hospital located in China from March 2017 to January 2018. Clinical information of patients was the basis for the further analyses of the isolates including antimicrobial susceptibility tests, identification of antibiotic resistance and virulence gene determinants, multilocus sequence typing (MLST), *XbaI*-macrorestriction by pulsed-field gel electrophoresis (PFGE). Selected isolates representing distinct resistance profiles and virulence phenotypes were screened for hypervirulence in a *Galleria mellonella* larvae infection model. In the course of the outbreak, the overall mortality rate of patients was 100% (*n* = 8) attributed to complications arising from CR-hvKP infections. All isolates except one (28/29, 96.6%) were resistant to multiple antimicrobial agents, and harbored diverse resistance determinants that included the globally prevalent carbapenemase *bla*_KPC−2_. Most isolates had hypervirulent genotypes being positive for 19 virulence-associated genes, including *iutA* (25/29, 86.2%), *rmpA* (27/29, 93.1%), *ybtA* (27/29, 93.1%), *entB* (29/29, 100%), *fimH* (29/29, 100%), and *mrkD* (29/29, 100%). MLST revealed ST11 for the majority of isolates (26/29, 89,7%). Infection assays demonstrated high mortality in the *Galleria mellonella* model with the highest LD_50_ values for three isolates (<10^5^ CFU/mL) demonstrating the degree of hypervirulence of these CR-hvKP isolates, and is discussed relative to previous outbreaks of CR-hvKP.

## Introduction

*Klebsiella pneumoniae* is a Gram-negative pathogen causing hospital-acquired infections, especially for immunocompromised patients in the intensive care units (ICU) (1–3). Variants called hypervirulent *K. pneumoniae* (hvKp) have evolved (4) which are becoming more prevalent. Diagnostically, hvKp tend to be hypermucoviscous due to increased secretion of capsular polysaccharide, and metabolically more robust through the acquisition of genes for more efficient iron acquisition from host tissues (2). Isolates of hvKP usually belong to sequence type ST23 and their capsule is usually of the fucosylated K1-type (5). There is also mounting evidence that hvKp secrete factors which impact the epithelia and microbiome, such as the cell-cycle modulating non-ribosomal peptide colibactin and the channel-forming bacteriocin microcin E492 (6, 7), respectively. These factors modify the host niche and might thereby contribute to the observed increase in virulence (6, 8). The significance of these features has been established in the measurement of virulence phenotype *in vitro* and *in vivo* (9–11), and whole genome sequence analyses document the range of genes for iron-acquisition, enhanced capsule secretion, and toxin secretion. These include iron-binding siderophores: aerobactin, yersiniabactin, and salmochelin, as well as TonB-dependent transporters for iron (Kfu), iron dicitrate (Fec), and ferric siderophores (FyuA) (7, 12).

Carbapenem-resistant *K. pneumoniae* (CRKP) are another global threat given that until recently carbapenems were last line drugs for *Klebsiella* infections (13–15). Carriage of the gene *bla*_KPC−2_ is the main molecular mechanism of carbapenem resistance in *K. pneumoniae* in Asia, especially in China, and carriage of the *bla*_KPC−2_ is mainly associated with strains of the clonal complex 258 (CC258). Among CC258, ST11 is the major sequence type (ST) from Asia, especially China (16).

The most abundant sequence type within this complex, ST258, emerged during the early to mid-2000s as an important human pathogen, descends from an ST11 ancestor which acquired a ~1,100 kb genomic region from an otherwise distantly related ST442-like *K. pneumoniae*(17–20). In China, the “ancestral” ST11 remains as a dominant clonal lineage of carbapenem-resistant *K. pneumoniae*, and forms the most common sequence type within CC258 in China (6, 13, 21–25).

Recently, a lineage of *K. pneumoniae* that simultaneously exhibits hypervirulence and carbapenem-resistance often refer to as “CR-hvKp” (12, 24, 26–28). These strains are of major concern: not only are they hypervirulent and multidrug-resistant, but they are also highly transmissible (24, 25, 29, 30). Sequence analysis of a recent fatal outbreak of ST11 CR-hvKP strains in a Chinese hospital revealed that the several isolates belonged to a single clone, and yet with slight variation in pulsed-field gel electrophoresis (PFGE) patterns between the strains suggestive of extraordinarily high mutation rates (24). The observations in that study underscore the urgent need for a better understanding of CR-hvKp.

In this study, we describe an outbreak of ST11 *K. pneumoniae* strains that are both carbapenem-resistant and hypervirulent, isolated from the ICU of the First Affiliated Hospital of Wenzhou Medical University. We sought to investigate the molecular and epidemiological features of these strains during the outbreak, especially on the characteristics of virulence factors and antimicrobial resistant determinants. The data from this outbreak confirmed that long-term carriage of CR-hvKp can give rise to in host evolution and an increase the repertoire of virulence factors carried. Hence, monitoring the nosocomial dissemination of CR-hvKp, providing effective strategies for anti-infective prophylaxis and outbreak management are essential.

## Materials and Methods

### Collection of *K. pneumoniae* Clinical Isolates From ICU

The First Affiliated Hospital of Wenzhou Medical University is a leading teaching, three-grade hospital in Wenzhou, a city in Zhejiang province, China. The hospital features 3,000 beds, 64 departments and 89 wards. It is one of the largest health care centers in south Zhejiang province, has responsibility for the medical care of an estimated 30 million population.

A total of 29 *K. pneumoniae* isolates were collected from eight patients from the ICU from March 2017 to January 2018. Patient information including age, gender, length of ICU stays, diagnosis, and outcomes is obtained from the Electronic Medical Records. The study and consent procedure were approved by the Ethical Committee of the First Affiliated Hospital of Wenzhou Medical University.

All isolates were isolated from various clinical specimens and identified as *K. pneumoniae* by VITEK-2 automated microbiology analyzer (bioMérieux, Marcy l'Etoile, France).

### Antimicrobial Susceptibility Testing (AST)

Minimal inhibitory concentrations (MICs) of antimicrobial agents (gentamicin, tobramycin, ampicillin, cefazolin, ceftazidime, ceftriaxone, imipenem, ertapenem, levofloxacin, and ciprofloxacin) were determined by the agar dilution method and interpreted according to the Clinical and Laboratory Standards Institute (document M100-S27). Furthermore, ASTs of tigecycline and polymyxin B were performed by the broth microdilution method and interpreted by the recommendation of the European Committee on Antimicrobial Susceptibility Testing clinical breakpoints (http://www.eucast.org). *Escherichia coli* ATCC25922 and *Pseudomonas aeruginosa* ATCC27853 served as the quality control strains for susceptibility testing.

### Molecular Detection of Antibiotic Resistance Determinant

Total DNA from each of the isolates was extracted from fresh bacterial colonies by using the Biospin Bacteria Genomic DNA Extraction kit (Bioflux, Tokyo, Japan). The presence of resistance determinants, including β-lactamase genes (*bla*_CTX−M−1_ group, *bla*_CTX−M−2_ group, *bla*_CTX−M−8_ group, *bla*_CTX−M−9_ group, *bla*_CTX−M−25_ group, *bla*_SHV_, *bla*_TEM_, *bla*_VEB_, *bla*_CMY_, and *bla*_DHA_), carbapenemase genes (*bla*_KPC_, *bla*_OXA−23_, *bla*_SPM_, *bla*_VIM_, *bla*_GES_, *bla*_NDM−1_, and *bla*_OXA−48_), plasmid-mediated quinolone resistance (PMQR) genes (*qnrA, qnrB, qnrC, qnrD, qnrS, qepA*, and *oqxA*), mutations in the GyrA and ParC proteins, the aminoglycoside resistance determinants [*aac(3')-Ia, aac(6')-Ib*, and *ANT(3')-Ia*], 16S-RMTase genes (*rmtB, rmtC, armA*), and the polymyxin resistance gene *mcr-1* were investigated by polymerase chain reaction (PCR) followed by sequencing. Primers used for PCR are shown in Table S2. The positive PCR products were sequenced by Beijing Genomics Institute Technology Co. Ltd. (Shanghai, China). Sequences alignments were completed by running BLAST at NCBI website (http://blast.ncbi.nlm.nih.gov/Blast.cgi).

### Detection of Virulence-Associated Features and Capsular Polysaccharide Synthesis (CPS) Genotyping

The string test was used for screening for the hypermucoviscous phenotype as previously reported (31). Those strains with viscous string over 5 mm were considered as hvKP (2). PCR amplification was used to identify the capsular serotype of 29 isolates, including K-serotype-specific alleles K1, K2, K5, K20, K54, and K57, which were the most frequent genotypes in hvKp (32). Presence of 19 virulence-associated genes, including *iutA, fepA, fyuA, iroN, iroD, rmpA, rmpA2, magA, kfuBC, wcaG, alls, ybtA, areA, uge, wabG, entB, fimH, mrkD*, and *iucA* were detected in 29 isolates. To detect the existence of pLVPK-like plasmid, specific primers of *repA, sopB, Lv049, Lv049* were designed based on the whole sequence previously reported (accession no. NC_005249.1). The specific primer sequences used in this study are listed in Table S1. The positive PCR products were sequenced by at Beijing Genomics Institute Technology Co. Ltd. (Shanghai, China). Nucleotide sequences alignments were compared completed by running BLAST at NCBI website (http://blast.ncbi.nlm.nih.gov/Blast.cgi).

### Molecular Epidemiology Analysis

Multilocus sequence typing (MLST) was performed for phylogenetic analyses according to the protocols on the Pasteur Institute MLST website for *K. pneumoniae* (https://bigsdb.pasteur.fr/klebsiella/klebsiella.html).

All strains were subjected to PFGE analysis. Strain DNA were digested through *XbaI* for 2 h at 37°C. Electrophoresis was conducted at 14°C for 18.5 h using the Bio-Rad^®^ CHEF-Mapper XA machine (Bio-Rad, California, USA). *Salmonella enterica* serotype Braenderup H9812 was used as the molecular marker (33). Reports-phylogenetic tree (UPGAMA) was constructed to determine the hierarchical representation of different linkage levels between the strains. Chromosomal DNA restriction patterns were interpreted using previously established guidelines (34), with a phylogenetic dendrogram analysis indicated at 85% demonstrating strain relatedness.

### *Galleria mellonella* Larvae Infection Assay

The virulence capability was evaluated through the commonly used *in vivo* infection model, the wax moth *Galleria mellonella*(7, 24, 35–37). According to the antimicrobial resistant profile and hypermucoviscous genotype reported previously, five strains were picked out for the further infection model. Two strains (Kp1 and Kp15) were resistant and hypermucoviscous as well, two strains (Kp4 and Kp21) were resistant but non-hypermucoviscous and one (Kp6) was susceptible and non-hypermucoviscous. The methods were described previously (35). In the present study, each infection group includes eight larvae and were injected with 10 μL of bacterial suspension with bacteria concentration ranging from 10^5^ to 10^7^ CFU per larva. As a control group, 10 μL PBS was injected in parallel. All larvae were placed in Petri-dishes and kept at 37°C in the dark. The number of dead larvae was recorded with notes on any melanization and lack of motility 24, 48, and 72 h after injection, respectively. All experiments were repeated in biological triplicate as described and results were not considered if two or more larvae in any of the control groups died. The survival curves were plotted by the GraphPad Prism 5.0 program and the Kaplan-Meier method was used for statistical analysis. *P* < 0.05 was considered statistically significant. For each isolate, the 50% lethal dose (LD_50_) was analyzed using the probit analysis program by SPSS 22.0 and the results were expressed as log_10_ LD_50_.

## Results

### Outbreak Description

**Figure 1:**
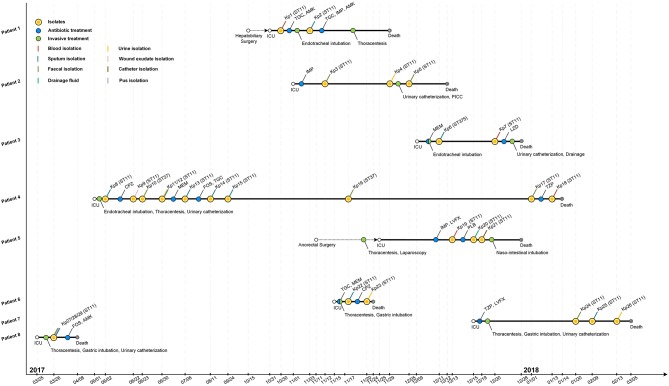
Epidemiology of the *K. pneumoniae* outbreak cases. Each horizontal line represents the timeline of one patient, with respect to treatments and isolation of tissue or fluid samples, with the abscissa axis showing the relevant dates. Isolates and the treatments of those patients are shown by colored dots. Different specimens are shown by colored lines. TGC, tigecycline; AMK, amikacin; IMP, imipenem; MEM, meropenem; LZD, linezolid; CPZ, cefoperazone; FOS, fosfomycin; TZP, piperacillin-tazobactam; LVFX, levofloxacin; PLB, polymyxin B; PICC, peripherally inserted central catheter.

From March 2017 to January 2018, eight patients (age range 13–69 years, ratio male-female 3:1) had fatal nosocomial infections during the period of hospitalization in the ICU. The eight patients died of respiratory failure, septic shock, and multiple organ failure (Table 1). All patients had submitted to invasive procedures (including surgery, endotracheal intubation, thoracentesis, urinary catheterization, laparoscopy, and gastric intubation) before the first *K. pneumoniae* was isolated in ICU (Figure 1). Patients diagnoses varied as pulmonary infection (4/8, 50%), post-operative infection (2/8, 25%), hemorrhagic infarct (1/8, 12.5%), and polytrauma (1/8, 12.5%). This event was characterized as an outbreak and attracted the attention of the hospital infection-control department.

**Table 1:** Characteristics of the eight patients and the isolated *K. pneumoniae* strains.

**Patient**	**Age**	**Length of stay (Days)**	**Diagnosis**	**Outcome**	**Isolate**	**Isolate date**	**Isolate type**	**ST type**	**PFGE cluster**
P1	50–60	28	Post-operation of cholelithiasis	Died	Kp1	31 October	Blood	11	E
					Kp2	3 November	Sputum	11	E
P2	50–60	31	Pulmonary infection	Died	Kp3	12 November	Fecal	11	E
			Brain infarct		Kp4	29 November	Urine	11	E
					Kp5	5 December	Fecal	11	E
P3	50–60	17	Polytrauma	Died	Kp6	11 December	Sputum	375	B
					Kp7	20 December	Blood	11	C
P4	60–70	228	Pulmonary infection	Died	Kp8	2 June	Sputum	11	E
			Brain stem tumor operation		Kp9	22 June	Wound exudate	11	E
					Kp10	23 June	Fecal	37	A
					Kp11	30 June	Urine	11	E
					Kp12	30 June	Sputum	11	E
					Kp13	6 July	Sputum	11	E
					Kp14	11 August	Sputum	11	E
					Kp15	24 September	Sputum	11	E
					Kp16	17 November	Fecal	37	A
					Kp17	1 January	Fecal	11	E
					Kp18	13 January	Blood	11	E
P5	60–70	34	Obstructive jaundice	Died	Kp19	13 December	Blood	11	D
			Tumor of duodenum		Kp20	15 December	Drainage fluid	11	D
					Kp21	19 December	Catheter	11	D
P6	10–20	10	Pulmonary infection	Died	Kp22	15 November	Sputum	11	E
					Kp23	17 November	Urine	11	E
P7	60–70	80	Hemorrhagic infarct	Died	Kp24	20 January	Fecal	11	D
					Kp25	9 February	Sputum	11	D
					Kp26	13 February	Urine	11	D
P8	60–70	15	Pulmonary infection	Died	Kp27	26 March	Fecal	11	E
					Kp28	26 March	Pus	11	E
					Kp29	26 March	Sputum	11	E

**Figure 2:**
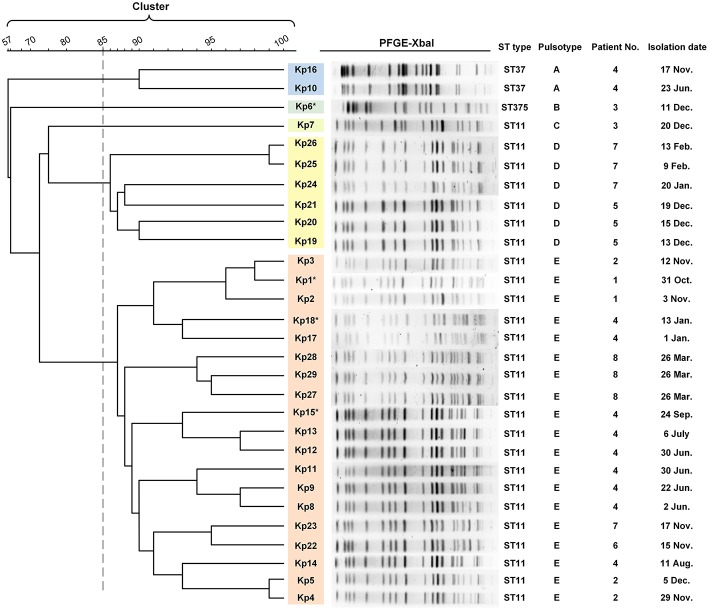
PFGE results and MLST typing for 29 *K. pneumoniae* isolates. Genomic DNA from each of the 29 *K. pneumoniae* isolates was digested using *XbaI* and the digests subjected to pulsed-field gel electrophoresis to generate diagnostic genomic DNA fragmentation fingerprints. The dendrogram of the PFGE profiles is clustered by the UPGAMA on the basis of the Dice similarity by the Quantity One software package 4.6. The sequences of seven housekeeping genes (*gapA, infB, mdh, pgi, phoE, rpoB*, and *tonB*) were analyzed for the purpose of MLST typing. PFGE results showed that 29 isolates were divided into five different PFGE clusters (A–E) as represented by different colors. Cluster D and E accounted for 20.6% (6/27) and 65.5% (19/27) of ST11 isolates, respectively, indicating that those strains were highly homologous. The two ST37 isolates belonged to the same cluster (cluster A). Kp6 (ST375) and Kp7 (ST11) formed separate monotypic clusters, cluster B and C, respectively. Asterisk indicates the string test of isolate was positive.

The retrospective clinical data showed that the first case (patient 8) was a male patient admitted to the ICU on 25th March 2017 with the diagnosis of pulmonary infection. On day first after hospitalization, three ST11 *K. pneumoniae* (Kp27, Kp28, and Kp29) isolates were retrieved from fecal, pus and sputum samples, respectively, and the patient died of fatal infection at day 15 (Figure 1, Table 1). The second case was patient 4, a 69-year-old man who underwent surgery for a brainstem tumor, and was admitted to the ICU on 1st June 2017 (Figure 1, Table 1). Patient 4 had the longest hospitalization length of stay (228 days), dating from June 2017 to January 2018. On 2nd June, *K. pneumoniae* was isolated from a sputum sample from patient 4. In the course of the ICU stay, patient 4 yielded 11 independent *Klebsiella* samples, with positive sputum samples recovered for 115 days (Figure 1). Administration of antibiotics (cefoperazone, meropenem, and tigecycline) did not resolve the ongoing respiratory infection. Two fecal samples from patient 4, in June and November, were also positive for *Klebsiella* (Figure 1) but were subsequently identified as a distinct sequence type (ST37, Figure 2), while other isolates belonged to ST11. Ultimately, an ST11 strain was isolated from the patient's blood in January 2018, immediately preceding death. Within this time frame, ST11 *K. pneumoniae* were isolated from other six patients in the ICU concurrently during this hospitalization period (Figure 1), indicating a potential of nosocomial transmission. The infection timeline is detailed in Figure 1.

To control this outbreak, all patients were placed in contact isolation. The patients were arranged in single rooms with disinfection of 2% chlorhexidine baths daily and the affected patients' rooms were disinfected by glutaraldehyde thoroughly. The staff members in the ICU were screened for signs and symptoms of infections daily. The outbreak was effectively controlled and besides these eight patients, no further infected patients were found in the ICU.

### Epidemiological Analysis and Virulence Characteristics

Multilocus sequence typing (MLST) analysis revealed that 26 isolates (26/29, 89.7%) retrieved from eight patients were identified as ST11 *K. pneumoniae*, indicating that this was the dominant ST type in these ICU deaths (Figure 2). In addition, three isolates belonged to either ST37 (patient 4, Kp10, Kp16; 6.9%) or ST375 (patient 3, Kp6; 3.4%). The similarities seen in the MLST phylogram indicated close relationships between some of the isolates. For example, the Kp1 isolate from the initial blood sample drawn from patient 1 was very closely related to the isolates Kp2 and Kp3 (Figure 2) drawn from both patient 1 and patient 2 in November (Figure 1). The subsequent urinary tract infection of patient 2 in December (Figure 1) was attributable to a distinct pair of ST11 isolates, Kp4, and Kp5 (Figure 2). The PFGE results showed that 29 isolates were divided into five different PFGE clusters (A to E). Cluster D and E accounted for 20.6% (6/27) and 65.5% (19/27) of ST11 isolates, respectively, indicating that these strains were highly homologous. The two ST37 isolates belonged to the same cluster (cluster A). Kp6 (ST375) and Kp7 (ST11) formed separate monotypic clusters, cluster B and C, respectively (Figure 2).

According to previous definition, the ST11 *K. pneumoniae* should be considered potentially hypervirulent (23, 24, 38). This, together with the high mortality rate (8/8 patients, 100%) in the outbreak led us to address the characteristics of these new isolates.

#### Extracellular Capsule

**Figure 3:**
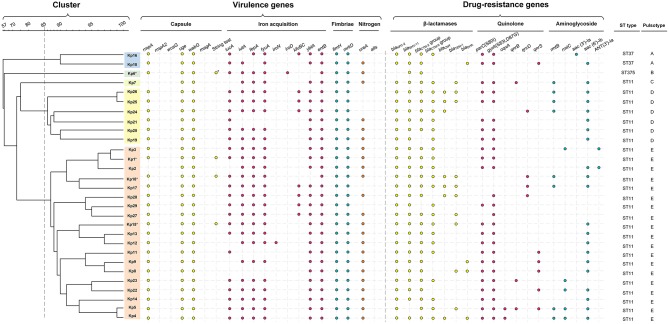
Gene profiles of *K. pneumoniae* isolates. Virulence genes and drug-resistance genes identified by PCR-based profiling are shown by colored dots, plotted against the phylogeny of the strains from the whole genome PFGE analysis shown in Figure 2. Asterisk indicates the capsule serotype was determined to be of K2 type. The relevant abbreviations are: *bla*, beta-lactamases (specific isoforms *bla*_KPC−2_ *, bla*_SHV−11_, *bla*_CTX−M−2_ group, *bla*_CTX−M−9_ group, *bla*_CMY−2_, *bla*_TEM−1_, *bla*_DHA_); mutations in *gyrA* (S83I, D87G) and *parC* (S80I), and the presence of *qnrB, qnrS*, and *oqxA* all reduce susceptibility to fluoroquinolones (42); *rmtB, rmtC* (encoding 16S rRNA methylases), *aac(3')-Ia, aac(6')-Ib*, and *ANT(3')-Ia* (encoding aminoglycoside-modifying enzymes) are all aminoglycoside resistance genes.

Only two isolates (Kp2, Kp7) lacked the transcriptional regulator of mucoid phenotype *rmp*A, a gene found responsible for upregulation of the synthesis of capsular polysaccharide (39). All of the isolates were negative for *rpmA2*, which has been reported as a trans-acting activator for the capsular polysaccharide biosynthesis (39). The *wzy*-like gene *magA* was involved in polysaccharide synthesis in K1 capsulated strains (40), and the biofilm-promoting fucose synthase *wcaG* (41) is required for decoration of the capsule polysaccharide with fucose. Both genes were absent from all isolates (Figure 3). While the ST11 strains were of indeterminate capsule type (i.e., not of the typical K1 capsule type) and only Kp6 (ST375) was identified as K2 capsule type (Figure 3). Interestingly, only four ST11 (Kp1, Kp6, Kp15, and Kp18) were string-test positive, which was correlated with a hypermucoviscous phenotype in previous studies (2, 43).

#### Iron Acquisition

All ST11 isolates except Kp8, Kp11, and Kp21 were positive for *iutA*, most isolates (23/29, 79.3%) were positive for *iucA*, indicating the existence of aerobactin uptake, the dominant component of the siderophore system involved in hypervirulence (44). All isolates were positive for *entB* and 25 isolates were positive for *fepA*, consistent with the use of the siderophore enterobactin for iron acquisition, and most ST11 isolates were also positive for *ybtA* (25/26) and *fyuA* (23/26), demonstrating that they could also use yersiniabactin for iron acquisition (Figure 3). Only some of the ST11 isolates had acquired the genes *kfuBC* encoding a ferric iron uptake ABC transporter. The ST375 isolate (Kp6) harbored siderophore uptake or biosynthesis genes: aerobactin (*iutA*), salmochelin (*iroD*), enterobactin (*fepA*), and yersiniabactin (*fyuA*), which are commonly associated with hypervirulence [Figure 3; (44)].

#### Fimbrial Adhesin

All ST11 isolates were positive for *fimH* and *mrkD* suggesting that they can express type 1 and type 3 fimbrial adhesion (45, 46), which mediate binding to the extracellular matrix and play an important role in biofilm formation (47).

#### Nitrogen Metabolism

All isolates were found to be negative for the *allS* transcriptional activator (Figure 3), suggesting they were incapable of allantoin utilization to compete for nitrogen access in host niches (48). Most of the isolates were positive for *ureA* (23/29), which enables the use of urea as a nitrogen source in host niches (49).

Besides, The pLVPK-like plasmid carrying capsular polysaccharides regulator genes (*rmpA* and *rmpA2*) and several siderophore gene clusters is known to contribute to the virulence of hvKp (24). Our results describe that while most isolates were positive for *iucA* (23/29, 79.3%), *repA* (28/29, 96.6%), *sopB* (28/29, 96.6%), *Lv049* (24/29, 82.8%), and *Lv204* (26/29, 89.7%), they were negative for *rmpA2*. Thus, consistent with a very recent study of ST23 *K. pneumoniae* in Japan (50), we inferred that the outbreak isolates might carry a modified version of the pLVPK plasmid.

### Carbapenem-Resistance and β-Lactamase Genes

The clinical break-point for carbapenem in the treatment of *Klebsiella* is 2 μg/mL (ertapenem) or 4 μg/mL (doripenem, imipenem and meropenem) (CLSI-M100, 2017). Isolates from patients 1 to 6, who did not respond to treatment with carbapenems, were confirmed *ex vivo* to be resistant to the carbapenems imipenem and ertapenem (MIC ranged from 8 to >128 μg/mL). With the exception of Kp6 (ST375), which did not display a multidrug-resistant phenotype, all other isolates carried the *bla*_KPC−2_ gene (Figure 3, Table S1). All isolates were negative for *bla*_NDM−1_ and *bla*_OXA−48_. Excluding Kp6, all isolates were resistant to β-lactams, including ampicillin, cefazolin, ceftazidime, and ceftriaxone (MIC > 64 μg/mL). The other β-lactamase genes detected, including *bla*_SHV−11_ (28 isolates), *bla*_CTX−M−2_ group (27 isolates), *bla*_CTX−M−9_ group (23 isolates), *bla*_TEM−1_ (10 isolates), *bla*_CMY−2_ (seven isolates), and *bla*_DHA_ (four isolates). These genes were distributed widely, but not uniformly, across the ST11 isolates (Figure 3, Table S1).

### Distribution of Other Drug-Resistance Mechanisms

Of the 29 *K. pneumoniae* isolates, 22 (75.9%) exhibited high-level resistance to the aminoglycoside gentamicin (32–>128 μg/mL), and 22 (75.9%) showed high-level resistance toward the aminoglycoside tobramycin (>128 μg/mL). This is consistent with observations that 24 isolates carried various aminoglycoside resistance determinants, including *aac(3')-Ia* and *aac(6')-Ib* each of which encodes aminoglycoside-modifying enzyme, *ANT(3')-Ia* (encoding an aminoglycoside adenyltransferase), *rmtB*, and *rmtC* each of which encodes a distinct 16S rRNA methylase that modifies ribosomes and causes resistance to aminoglycosides (Figure 3).

Most (27/29, 93.1%) isolates were resistant to the quinolones levofloxacin and ciprofloxacin (MIC ranged from 16 to 128 μg/mL), except for Kp16, which was judged intermediate in resistance to levofloxacin and ciprofloxacin (MIC = 4 μg/L), and Kp6, which was susceptible to both of them (MIC ≤ 0.25 μg/mL). Resistance to fluoroquinolones was largely due to point mutations; 25 isolates carried missense quinolone-resistance mutations in the DNA replication machinery, including *parC* (24 isolates: S80I in DNA topoisomerase IV) and *gyrA* (25 isolates: S83I, D87G in DNA gyrase). In addition, plasmid-mediated quinolone resistance determinants *qnrB, qnrD, qnrS*, and *oqxA* were also determined in several isolates (Figure 3).

Polymyxin-resistance was measurable in two of the isolates, Kp12 (patient 4, ST11) and Kp21 (patient 5, ST11), but not in any others. Nevertheless, these two strains were negative for *mcr-1*, the most prominent cause of polymyxin resistance in China (51, 52). However, other resistance mechanism such as modifications of two-component systems (PmrA/PmrB and PhoP/PhoQ) and inactivation of the *mgrB* gene (53) might be present, but would require to be further investigated. Interestingly, patient 5 did not respond to polymyxin treatment, despite Kp19 (ST11) being deemed polymyxin-sensitive *ex vivo* (Table S3). Similarly, for the glycylcycline antibiotic tigecycline, patients 1, 4, and 6 did not respond to intravenous treatment with the antibiotics, despite all isolates being deemed sensitive to tigecycline *ex vivo* (Table S3).

### Hypervirulence Measurements Through the Death of *G. mellonella* Larvae

Five isolates were collected based on the antimicrobial resistance profile and hypermucoviscous phenotype: Kp6 (patient 3, ST375, hypermucoviscous, antibiotic-sensitive), Kp1 (patient 1, ST11, hypermucoviscous, carbapenem-resistant, *bla*_KPC−2_ positive), and Kp15 (patient 4, ST11, hypermucoviscous, carbapenem-resistant, *bla*_KPC−2_ positive), Kp4 (patient 2, ST11, non-hypermucoviscous, carbapenem-resistant, *bla*_KPC−2_ positive), and Kp21 (patient 5, ST11, non-hypermucoviscous, carbapenem-resistant, *bla*_KPC−2_ positive). Among the five selected strains, the Kp1 isolate from the initial blood sample drawn from patient 1 is hypermucoviscous as determined by string-test and resistant to beta-lactams, carbapenem and quinolones. During the outbreak this strain was most likely responsible for the infection of patient 1 possibly transmitted to patient 2 (Figure 1, Table S3). The isolate Kp6 was ST375, showed a positive string test as well as acquisition of genes for *iutA*, which predicted it could be hypervirulent. Kp21 was isolated from a urinary catheter of patient 5 before expiration. From our analysis it was determined to be non-hypermucoviscous, but extremely drug-resistant. It was therefore of interest to determine the virulence phenotypes of Kp1, Kp6, and Kp21. In addition, Kp4 and Kp15 were selected randomly from each of the two groups with hypermucoviscous phenotype and non-hypermucoviscous phenotype.

**Figure 4:**
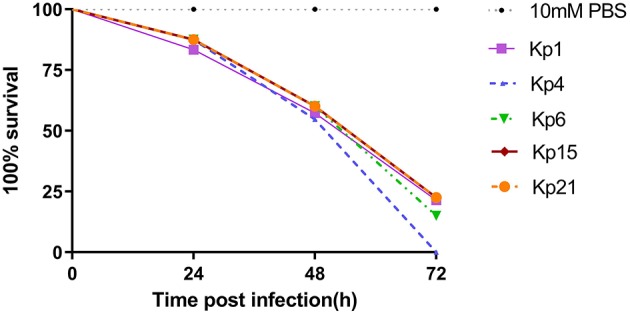
*K. pneumoniae* infection of *G. mellonella* larvae induces time-dependent lethality. The effect of 1 × 10^6^ colony-forming units (CFU) of each *K pneumoniae* strain on survival was observed in *G. mellonella*. The strains were divided into three groups according the hypermucoviscous and resistance phenotypes. High-level drug resistant and hypermucoviscous (Kp1, Kp15). High-level drug resistant and non-hypermucoviscous (Kp4, Kp21). Drug sensitive but hypermucoviscous (Kp6). Larvae were injected with PBS or with 10^6^ CFU of Kp1, Kp4, Kp6, Kp15, and Kp21. The survival was monitored over 72 h post-infection. Mortality of larvae infected with Kp1, Kp4, Kp6, Kp15, and Kp21 were time-dependent.

All isolates caused a time-dependent death of the larvae. When the dose of bacteria was altered (10^5^, 10^6^, 10^7^ CFU), *K. pneumoniae*-induced lethality was found to be dependent on the inoculum density (Figure S1). After 72 h of infection, the mortality of the larvae infected with 10^7^ CFU bacterial suspension was higher than that infected with 10^5^ CFU bacterial suspension. The observed mortality of the larvae was higher than 60% after 72 h (Figure 4) with the most virulent being Kp4 (100% mortality), Kp21 (100% mortality), and Kp6 (75% mortality). There was no significant difference among these groups (*P* > 0.05).

The recent assessment of a range of *K. pneumoniae* isolates suggests the parameters for the *Galleria* model to define hypervirulence, based on a calculation of LD_50_ value (37). In Shi's study, isolates of *K. pneumoniae* generating an LD_50_ <5.06 log_10_ CFU/ml were defined as hypervirulent. According to that study, we calculated LD_50_ for Kp1 (5.324 log_10_ CFU/ml), Kp4 (5.755 log_10_ CFU/ml), Kp6 (4.954 log_10_ CFU/ml), Kp15 (5.017 log_10_ CFU/ml), and Kp21 (4.266 log_10_ CFU/ml).

## Discussion

Our study reported an outbreak of ST11 *K. pneumoniae* in the ICU of a teaching hospital where multiple isolates showed highly multiple-drug resistance phenotypes including carbapenem-resistance. Sampling from patients showed continuing colonization with *K. pneumoniae*. Isolates were taken under a surveillance protocol aimed at patient care, which means sampling bias interferes with drawing strong conclusions on the extent of in host evolution vs. transmission and re-infection by multiple ST11 *K. pneumoniae* strains. Nonetheless, several important points can be made from the data gathered from this ICU outbreak.

Firstly, this study is consistent with observations of long-term colonization by carbapenem-resistant *K. pneumoniae* reported by three other studies of hospital-acquired infection (54–56). As the ICU outbreak reported in the United States of America, two patients who survived the outbreak remained colonized by carbapenem-resistant *K. pneumoniae* for up to 4 years. A single clone was detected in samples from various sites (throat, groin, rectum) of the patients, and the documented characteristics of in host evolution over these years suggested recombination and gene loss events, particularly affecting the plasmids carried by the long-term colonizing *K. pneumonia* (56). In this outbreak in the First Affiliated Hospital in Wenzhou, patient 4 remained colonized by carbapenem-resistant *K. pneumoniae*, with positive sputum samples recovered for 115 days.

Secondly, most *K. pneumoniae* (26/29, 89.7%) in this outbreak were assigned to ST11 lineage which was reported as being prevalent in the wide spread of KPC-producing *K. pneumoniae* in China (13). In early 2011, a nosocomial outbreak of *bla*_KPC−2_ positive ST11 CRKP infection was observed in the north of China (57). Subsequently, several outbreaks of *bla*_KPC−2_ positive ST11 CRKP were reported in multiple hospitals in China (58–61), echoing the prevalence of those stains in the mainland of China. In our study, all ST11 CRKP harbored *bla*_KPC−2_ and *bla*_SHV−11_, and most were also positive for *bla*_TEM−1_ and *bla*_CTX−M_, consistent with previous reports. ST11 CR-hvKP has been recently reported elsewhere in China, causing fatal infections and high mortality in other hospitals (24, 38, 61, 62).This seemingly recent increase in occurrence of the ST11 CR-hvKP in various Chinese hospitals is of grave concern, as it indicates either the presence of these strains within the community, or the high potential of ST11 *K. pneumoniae* to repeatedly yet independently acquire hypervirulence genes in the distinct hospitals to cause serious outbreaks. The isolates presented in our study carried more resistance determinates in their genotypes, and showed correspondingly more multi-drug resistant phenotypes, compared to other reports in the literature. This indicates that increasingly serious threats are emerging.

Thirdly, our study highlights some short-comings in real-time diagnostics of drug-resistant phenotypes and genotypes in the case of *K. pneumoniae* detection. Technologically, it is now feasible to apply partial-targeted or whole genome sequencing (WGS) to patient samples in order to determine drug resistance profiles. However, there were multiple instances in this outbreak where isolated bacteria were sensitive to the antibiotic used *ex vivo*, nevertheless, the infection caused by these isolates did not respond to antibiotic treatments effectively *in vivo*. For example, in the case of patient 4 administration of antibiotics including tigecycline was ineffective in treating the respiratory infection, despite the observation that *ex vivo*, the isolates from patient 4 were tigecycline-sensitive. Another example was in the case of patient 5, polymyxin B also failed to treat the infection although the isolates was sensitive *ex vivo*. For future outbreaks, introducing WGS analysis should be considered as it can identify pathogenic virulence factors, diagnostic for hvKP variants guiding specific hospital outbreak control measures and treatment plans to be actioned (24, 60, 62, 63). For instance, due to the high transmission nature of hvKP (24, 64), time-sensitive treatment and strict isolation measures should be performed immediately after detection to prevent further possible transmission reducing. Furthermore, as hvKP often present as abscesses, drainage of these sites of infection should be considered (2), and strict glycemic control may help clinicians to reduce the possibility of septic metastatic complications (43).

Finally, with the emergence of drug-resistances in hypervirulent strains of *K. pneumoniae*, it is incumbent on the community to have clearer definitions of the term “hypervirulence”. The accepted clinical definition of hvKP was based on manifestations that include liver abscesses, metastatic meningitis and endophthalmitis (2). In some studies, hypervirulent strains were identified by their hypermucoviscous phenotype through string test (26, 38, 65). Alternatively, hypervirulence gene signatures have been developed through genome-wide association studies that mostly identified an increased capability of iron acquisition and likely production of a hypermucoviscous phenotype (2, 6–8, 11, 12, 66). Ultimately, the definition of a hypervirulent phenotype is a greatly increased level of virulence. Larvae from *Galleria mellonella*, a wax moth, are becoming more widely used to study the pathogenesis of *K. pneumoniae* infections and the efficacy of antimicrobial drugs and phage-based therapies (24, 35–37, 67, 68). It is an experimentally tractable model and the similarities between these insects and vertebrate innate immune responses make it suitable for screening and comparative analysis of bacteria isolated from human patients (69). The results presented here show that the *K. pneumoniae* infection of *G. mellonella* larvae induced dose-dependent and time-dependent lethality. Importantly, even with strains that covered a range of drug-resistance and hypermucoviscous phenotypes, similarly high mortality rates were observed in the animal model of infection, with no significant difference between these isolates. This data suggests that hypervirulence is not directly predictable from genetic signatures indicative of hypermucoviscosity or iron-acquisition phenotypes. Our data support the proposition that “hypervirulent” could be defined through calculated LD_50_ value drawn from a standardized *Galleria* model assay (37). The threshold value used to denote hypervirulence should be addressed by the *Klebsiella* research community.

In conclusion, our study described the outbreak of ST11 *K. pneumoniae* that exhibited hypervirulence and multidrug resistance in one of the largest comprehensive hospitals in China. With the prevalence of such CR-hvKp, severely challenges have appeared in clinical treatments and pose a substantial threat to the world's population. Sustained efforts are required to limit the transmission of these strains, and prevent future outbreaks.

## Data Availability

The raw data supporting the conclusions of this manuscript will be made available by the authors, without undue reservation, to any qualified researcher.

## Author Contributions

XZ, YaZ, WB, and YiZ contributed to the design of the experiments. XZ, YiZ, and YaZ performed the experiment. TL and YaZ wrote the initial draft of the manuscript. XZ, YiZ, LC, and YaZ contributed to the acquisition, analysis, and interpretation of the data included in this manuscript. VT, AR, JL, RS, SZ, TZ, and JC revised the manuscript. JW revised the figures. All authors approve of the final manuscript being submitted and agree to be accountable for the work detailed in the submitted manuscript.

### Conflict of Interest Statement

The authors declare that the research was conducted in the absence of any commercial or financial relationships that could be construed as a potential conflict of interest.
