# Supplementary material for: An Outbreak of Carbapenem-Resistant and Hypervirulent Klebsiella pneumoniae in an Intensive Care Unit of a Major Teaching Hospital in Wenzhou, China
Source: Front Public Health. 2019 Aug 19;7:229. doi: 10.3389/fpubh.2019.00229 (PMC6736603; doi:10.3389/fpubh.2019.00229)
Supplement: Supplementary file 1 [file Data_Sheet_1.pdf]

Supplementary Material

1 Supplementary Figures and Tables

1.1 **Table S1.** List of oligonucleotide primers used in amplification of virulence-associated genes and capsular serotyping detection.

**Gene/marker**	**Primer Sequence (5’---3’)**	**Tm (°C)**	**Amplicon size (bp)**	**Reference**
magA	F: GGTGCTCTTTACATCATTGC	58	1283	(1)
	R: GCAATGGCCATTTGCGTTAG			
iutA	F: GTTGCGATTCTACCCGTTCC	57	248	this study
	R: GCCCGGTGGTGTAAATCTTC			
fepA	F: CCTGCCGATTGATTTCCTCG	57	226	this study
	R: CGTTACGATGGTGCTGTCAG			
fyuA	F: ATATGGCAAAAGCGCTCAGG	57	204	this study
	R: GGGTTAATCATGTCGCCGTC			
iroD	F: GCATAGGCGGATACGAACAT	58	556	this study
	R: CACAGGGCAATTGCTTACCT			
iroN	F: GGCTACTGATACTTGACTATTC	58	992	(2)
	R: CAGGATACAATAGCCCATAG			
kfuBC	F: GAAGTGACGCTGTTTCTGGC	58	797	(3)
	R: TTTCGTGTGGCCAGTGACTC			
rmpA	F: ACTGGGCTACCTCTGCTTCA	53	535	(4)
	R: CTTGCATGAGCCATCTTTCA			
wcaG	F: GGTTGGKTCAGCAATCGTA	58	169	

	R: ACTATTCCGCCAACTTTTGC			(5)
alls	F: CCGAAACATTACGCACCTTT	58	508	(3)
	R: ATCACGAAGAGCCAGGTCAC			
ybtA	F: ATGACGGAGTCACCGCAAAC	55	960	(6)
	R: TTACATCACGCGTTTAAAGG			
ureA	F: GACAAGCTGTTGCTGTTTACC	58	270	(7)
	R: CGGGTTGTGAACGGTGAC			
uge	F: GATCATCCGGTCTCCCTGTA	53	534	(8)
	R: TCTTCACGCCTTCCTTCACT			
wabG	F: CGGACTGGCAGATCCATATC	58	683	(9)
	R: ACCATCGGCCATTTGATAGA			
entB	F: ATTTCCTCAACTTCTGGGGC	56	371	(1)
	R: AGCATCGGTGGCGGTGGTCA			
fimH	F: TGCTGCTGGGCTGGTCGATG	62	688	(1)
	R: GGGAGGGTGACGGTGACATC			
mrkD	F: CCACCAACTATTCCCTCGAA	43	226	(4)
	R: ATGGAACCCACATCGACATT			
iucA	F: ATCCACCAGCAGGTTTTCAC	60	591	this study
	R: ATCCACCAGCAGGTTTTCAC			
rmpA2	F: CGTATGAAGGCTCGATGGAT	60	639	this study
	R: TGTGCACCATTTTTCATCAG			
repA	F: GATATCCCGAAGTTGCTCCA	60	545	this study
	R: ACGTTAAGATCACCGGTTCG			
sopB	F: CCGAAGATGCTCTGGATGAT	60	560	

	R: TGATGCGGAAGATTGTTTCA			this study
LV049	F: TGATGCGGAAGATTGTTTCA<br>R: GATCCAGCTGAAGTCGGTGT	60	509	this study
LV206	F: TCCACAATCCTGCCTTTTTC<br>R: GGCTCATCAGGGTCAGGTAA	60	557	this study
K1 (Capsular serotype)	F: GGTGCTCTTTACATCATTGC<br>R: GCAATGGCCATTTGCGTTAG	54	1283	(4)
K2 (Capsular serotype)	F: GACCCGATATTCATACTTGACAGAG<br>R: CCTGAAGTAAAATCGTAAATAGATGGC	58	641	(10)
K5 (Capsular serotype)	F: TGGTAGTGATGCTCGCGA<br>R: CCTGAACCCACCCCAATC	58	280	(10)
K20 (Capsular serotype)	F: CGGTGCTACAGTGCATCATT<br>R: GTTATACGATGCTCAGTCGC	58	741	(10)
K54 (Capsular serotype)	F: CATTAGCTCAGTGGTTGGCT<br>R: GCTTGACAAACACCATAGCAG	58	881	(10)
K57 (Capsular serotype)	F: CTCAGGGCTAGAAGTGTCAT<br>R: CTCAGGGCTAGAAGTGTCAT	58	1037	(10)

References

1. E. Candan and N. Aksöz: *Klebsiella pneumoniae*: characteristics of carbapenem resistance and virulence factors. *Acta Biochim. Pol.*, 62(4), 867-74 (2015). doi: 10.18388/abp.2015_1148.

2. P.-F. Hsieh, T.-L. Lin, C.-Z. Lee, S.-F. Tsai and J.-T. Wang: Serum-induced iron-acquisition systems and TonB contribute to virulence in *Klebsiella pneumoniae* causing primary pyogenic liver abscess. *Journal of Infectious Diseases*, 197(12), 1717-1727 (2008). doi: 10.1086/588383.

3. W. Yu, et al.: Comparison of prevalence of virulence factors for *Klebsiella pneumoniae* liver abscesses between isolates with capsular K1/K2 and non-K1/K2 serotypes. *Diagn. Microbiol. Infect. Dis.*, 62(1), 1-6 (2008). doi: 10.1016/j.diagmicrobio.2008.04.007

4. R. Wasfi, W. Elkhatib and H. Ashour: Molecular typing and virulence analysis of multidrug resistant *Klebsiella pneumoniae* clinical isolates recovered from Egyptian hospitals. *Sci Rep*, 6, 38929 (2016). doi: 10.1016/j.diagmicrobio.2008.04.007.

5. J. F. Turton, C. Perry, S. Elgohari and C. V. Hampton: PCR characterization and typing of *Klebsiella pneumoniae* using capsular type-specific, variable number tandem repeat and virulence gene targets. *Journal of medical microbiology*, 59(5), 541-547 (2010). doi: 10.1099/jmm.0.015198-0.

6. S. Zhang, et al.: Phenotypic and Genotypic Characterization of Isolated From Retail Foods in China. *Front Microbiol*, 9, 289 (2018). doi: 10.1016/j.ijfoodmicro.2019.05.021

7. L. Zhan, et al.: Outbreak by hypermucoviscous *Klebsiella pneumoniae* ST11 isolates with carbapenem resistance in a tertiary hospital in China. *Frontiers in cellular and infection microbiology*, 7, 182 (2017). doi:10.3389/fcimb.2017.00182.

8. W. Yu, et al.: Association between rmpA and magA genes and clinical syndromes caused by *Klebsiella pneumoniae* in Taiwan. *Clin. Infect. Dis.*, 42(10), 1351-8 (2006). doi: 10.3389/fcimb.2017.00182.

9. W.-H. Lin, et al.: Clinical and microbiological characteristics of *Klebsiella pneumoniae* isolates causing community-acquired urinary tract infections. *Infection*, 38(6), 459-464 (2010). doi: 10.1007/s15010-010-0049-5.

10. J. Turton, C. Perry, S. Elgohari and C. Hampton: PCR characterization and typing of *Klebsiella pneumoniae* using capsular type-specific, variable number tandem repeat and virulence gene targets. *J. Med. Microbiol.*, 59(Pt 5), 541-7 (2010). doi: 10.1099/jmm.0.015198-0.

1.2 **Table S2.** List of oligonucleotide primers used in amplification of resistance genes.

Gene	Primer Sequence (5’---3’)	Tm (°C)	Amplicon size (bp)	Reference
bla$_{\text{CTX-M-1}}$ group	F: GGTTAAAAAATCACTGCGTC<br>R: TTGGTGACGATTTTAGCCGC	48	825-863	(1)
bla$_{\text{CTX-M-2}}$ group	F: ATGATGACTCAGAGCATTCG<br>R: TGGGTTACGATTTTCGCCGC	50	827-865	(1)
bla$_{\text{CTX-M-8}}$ group	F: ATGGTACGACGAATGATATC<br>R: TAATCATACAGAAGTCGCAG	48	246	(2)
bla$_{\text{CTX-M-9}}$ group	F: ATGGTGACAAAGAGAGTGCA<br>R: CCCTTCGGCGATGATTCTC	50	863-826	(1)
bla$_{\text{CTX-M-25}}$ group	F: ATGATGAGAAAAAGCGTAAG<br>R: TTAATAACCGTCGGTGAC	50	869	(3)
bla$_{\text{CTX-M-65}}$	F: GCAGTACAGCGACAATACCG<br>R: TATCACCCACAGTCCACGAC	50	320	This study
bla$_{\text{SHV}}$	F: AACGCTTTCCCATGATGAGC<br>R: CGCCTCATTCAGTTCCGTTT	55	322	This study
bla$_{\text{TEM}}$	F: GGGAACCGGAGCTGAATGAA<br>R: CAGTGCTGCAATGATACCGC	59	254	This study
bla$_{\text{VEB}}$	F: ACGGTAATTTAACCAGATAGG<br>R: ACCCGCCATTGCCTATGAGCC	46	970	(4)
bla$_{\text{CMY}}$	F: TGGCCAGAACTGACAGGCAAA<br>R: TTTCTCCTGAACGTGGCTGGC	60	462	(5)
bla$_{\text{DHA}}$	F: AACTTTCACAGGTGTGCTGGGT<br>R: CCGTACGCATACTGGCTTTGC	50	405	(6)

bla$_{NDM-1}$	F: TCTCGACATGCCGGGTTTCGG<br>R: ACCGAGATTGCCGAGCGACTT	57	475	(7)
bla$_{KPC-2}$	F: CGCCAATTTGTTGCTGAAGG<br>R: CATAGTCATTTGCCGTGCCA	55	341	This study
bla$_{OXA-23}$	F: AAGCCGCGCAAATACAGAAT<br>R: ATCCATTGCCCAACCAGTCT	50	463	This study
bla$_{OXA-48}$	F: CGCATCTTGTTGTCCAAGTG<br>R: TCGAGCATCAGCATTTTGTC	52	1012	(8)
bla$_{SPM}$	F: CCTACAATCTAACGGCGACC<br>R: TCGCCGTGTCCAGGTATAAC	55	649	(9)
bla$_{VIM}$	F: GGTCTCATTGTCCGTGATGGTGATGAG<br>R: CTCGATGAGAGTCCTTCTAGAG	50	271	(10)
bla$_{GES}$	F: AGAATTGACTCAGGCACCGA<br>R: TGTGTTGTCGCTCATCTCCT	59	864	This study
qnrA	F: ATTTCTCACGCCAGGATTTG<br>R: GATCGGCAAAGGTTAGGTCA	48	627	(11)
qnrB	F: GATCGTGAAAGCCAGAAAGG<br>R: ACGATGCCTGGTAGTTGTCC	45	469	(11)
qnrC	F: GGGTTGTACATTTATTGAATCG<br>R: CACCTACCCATTTATTTTCA	50	307	(12)
qnrD	F: CGAGATCAATTTACGGGGAATA<br>R: AAC AAG CTG AAG CGC CTG	50	533	(13)
qnrS	F: ACGACATTCGTCAACTGCAA<br>R: TAAATTGGCACCCTGTAGGC	53	417	(14)
qepA	F: GAGATCATCATCACCAGCGC	58	573	This study

	R: ACCTTCCACGAGATCAAGCA			
oqxA	F: CACTATCAAAACCTCGCCCG	58	396	This study
	R: CCCAGGATTCAACCCCTTCT			
gyrA	F: CGCGTACTATACGCCATGAACGTA	55	441	(15)
	R: ACCGTTGATCACTTCGGTCAGG			
parC	F: ATGTACGTGATCATGGACA	55	389	(16)
	R: ATTCGGTGTAACGCATGG			
AAC (3')-Ia	F: ATGGGCATCATTCGCACATGTAGG	58	465	(17)
	R: TTAGGTGGCGGTACTTGGGTC			
AAC (6')-Ib	F: TTGCGATGCTCTATGAGTGGCTA	57	482	(17)
	R: CTCGAATGCCTGGCGTGTTT			
rmtB	F: CCCAAACAGACCGTAGAGGC	50	585	(18)
	R: CTCAAACTCGGCGGGCAAGC			
rmtC	F: TGGTGGGATCGATGGGAAAA	50	271	This study
	R: GCCAGCCTCCGTAAAGAATG			
armA	F: AGGTTGTTTCCATTTCTGAG	50	591	(18)
	R: TCTCTTCATTCCCTTCTCC			
ANT(3')-Ia	F: TTGCAGGTATCTTCGAGCCA	50	433	This study
	R: TTGCCGACTACCTTGGTGAT			
mcr-1	F: GCTCGGTCAGTCCGTTTG	55	1586	(19)
	R: GAATGCGGTGCGGTCTTT			

References

1. M. Saladin, et al.: Diversity of CTX-M beta-lactamases and their promoter regions from *Enterobacteriaceae* isolated in three Parisian hospitals. *FEMS Microbiol Lett*, 209(2), 161-8 (2002). doi:10.1111/j.1574-6968.2002.tb11126.x
2. C. J. Munday, J. Xiong, C. Li, D. Shen and P. M. Hawkey: Dissemination of CTX-M type beta-lactamases in *Enterobacteriaceae* isolates in the People's Republic of China. *Int J Antimicrob Agents*, 23(2), 175-80 (2004). doi:10.1016/j.ijantimicag.2003.07.004
3. M. Cartelle, et al.: Characterisation of the first CTX-M-10-producing isolate of *Salmonella enterica* serotype Virchow. *Clin Microbiol Infect*, 12(3), 285-7 (2006). doi:10.1111/j.1469-0691.2005.01300.x
4. J. Schlesinger, et al.: Extended-spectrum beta-lactamases among Enterobacter isolates obtained in Tel Aviv, Israel. *Antimicrob Agents Chemother*, 49(3), 1150-6 (2005). doi:10.1128/AAC.49.3.1150-1156.2005
5. J. Kim, et al.: CTX-M and SHV-12 beta-lactamases are the most common extended-spectrum enzymes in clinical isolates of *Escherichia coli* and *Klebsiella pneumoniae* collected from 3 university hospitals within Korea. *FEMS Microbiol Lett*, 245(1), 93-8 (2005). doi:10.1016/j.femsle.2005.02.029
6. F. J. Perez-Perez and N. D. Hanson: Detection of plasmid-mediated AmpC beta-lactamase genes in clinical isolates by using multiplex PCR. *J Clin Microbiol*, 40(6), 2153-62 (2002). doi:10.1128/jcm.40.6.2153-2162.2002
7. L. Hidalgo, et al.: Association of the novel aminoglycoside resistance determinant RmtF with NDM carbapenemase in Enterobacteriaceae isolated in India and the UK. *J Antimicrob Chemother*, 68(7), 1543-50 (2013). doi:10.1093/jac/dkt078
8. L. Ma, et al.: Emergence of OXA-48-Producing *Klebsiella pneumoniae* in Taiwan. *PLoS One*, 10(9), e0139152 (2015). doi:10.1371/journal.pone.0139152
9. G. Yu, et al.: First report of novel genetic array *aacA4-blaIMP-25-oxa30-catB3* and identification of novel metallo-beta-lactamase gene *bla*$_{IMP2}$5: A Retrospective Study of antibiotic resistance surveillance on *Psuedomonas aeruginosa* in Guangzhou of South China, 2003-2007. *Microb Pathog*, 95, 62-67 (2016). doi:10.1016/j.micpath.2016.02.021
10. F. M. Kaczmarek, F. Dib-Hajj, W. Shang and T. D. Gootz: High-level carbapenem resistance in a *Klebsiella pneumoniae* clinical isolate is due to the combination of bla(ACT-1) beta-lactamase production, porin OmpK35/36 insertional inactivation, and down-regulation of the phosphate transport porin phoe. *Antimicrob Agents Chemother*, 50(10), 3396-406 (2006). doi:10.1128/AAC.00285-06
11. A. Robicsek, et al.: Fluoroquinolone-modifying enzyme: a new adaptation of a common aminoglycoside acetyltransferase. *Nat Med*, 12(1), 83-8 (2006). doi:10.1038/nm1347

12. H. B. Kim, et al.: Prevalence of plasmid-mediated quinolone resistance determinants over a 9-year period. *Antimicrob Agents Chemother*, 53(2), 639-45 (2009). doi:10.1128/AAC.01051-08

13. L. M. Cavaco, H. Hasman, S. Xia and F. M. Aarestrup: *qnrD*, a novel gene conferring transferable quinolone resistance in *Salmonella enterica* serovar Kentucky and Bovismorbificans strains of human origin. *Antimicrob Agents Chemother*, 53(2), 603-8 (2009). doi:10.1128/AAC.00997-08

14. A. Robicsek, D. F. Sahm, J. Strahilevitz, G. A. Jacoby and D. C. Hooper: Broader distribution of plasmid-mediated quinolone resistance in the United States. *Antimicrob Agents Chemother*, 49(7), 3001-3 (2005). doi:10.1128/AAC.49.7.3001-3003.2005

15. S. Brisse and J. Verhoef: Phylogenetic diversity of *Klebsiella pneumoniae* and *Klebsiella oxytoca* clinical isolates revealed by randomly amplified polymorphic DNA, *gyrA* and *parC* genes sequencing and automated ribotyping. *Int J Syst Evol Microbiol*, 51(Pt 3), 915-24 (2001). doi:10.1099/00207713-51-3-915

16. Y. S. Nam, et al.: Investigation of mutation distribution in DNA gyrase and topoisomerase IV genes in ciprofloxacin-non-susceptible *Enterobacteriaceae* isolated from blood cultures in a tertiary care university hospital in South Korea, 2005-2010. *Int J Antimicrob Agents*, 41(2), 126-9 (2013). doi:10.1016/j.ijantimicag.2012.10.004

17. B. C. Haldorsen, G. S. Simonsen, A. Sundsfjord, O. Samuelsen and R. Norwegian Study Group on Aminoglycoside: Increased prevalence of aminoglycoside resistance in clinical isolates of *Escherichia coli* and *Klebsiella* spp. in Norway is associated with the acquisition of AAC(3)-II and AAC(6')-Ib. *Diagn Microbiol Infect Dis*, 78(1), 66-9 (2014). doi:10.1016/j.diagmicrobio.2013.10.001

18. L. Ma, et al.: Widespread dissemination of aminoglycoside resistance genes *armA* and *rmtB* in *Klebsiella pneumoniae* isolates in Taiwan producing CTX-M-type extended-spectrum beta-lactamases. *Antimicrob Agents Chemother*, 53(1), 104-11 (2009). doi:10.1128/AAC.00852-08

19. J. Quan, et al.: Prevalence of mcr-1 in *Escherichia coli* and *Klebsiella pneumoniae* recovered from bloodstream infections in China: a multicentre longitudinal study. *Lancet Infect Dis*, 17(4), 400-410 (2017). doi:10.1016/S1473-3099(16)30528-X

1.3 **Table S3.** Antimicrobial susceptibility and hypermucoviscous phenotype

Isolates (*-hvKp)	MIC (μg/mL)												ST type	PFGE cluster
	Aminoglycosides		Beta-lactams				Carbapenems		Quinolones		Others			
	GEN	TOB	AMP	CEZ	CAZ	CTR	IMP	ETP	LVFX	CPFX	PLB	TGC		
Kp1*	0.5	0.5	>128	>128	>128	>64	64	>64	32	64	0.25	2	11	E
Kp2	32	0.5	>128	>128	>128	>64	16	>64	32	64	0.5	2	11	E
Kp3	>128	>128	>128	>128	>128	>64	32	>64	128	>64	1	2	11	E
Kp4	>128	>128	>128	>128	>128	>64	32	>64	64	>64	0.125	2	11	E
Kp5	>128	>128	>128	>128	>128	>64	32	>64	64	64	0.25	2	11	E
Kp6*	1	0.5	64	8	4	1	<0.25	>64	0.25	<0.25	0.5	2	375	B
Kp7	>128	>128	>128	>128	64	>64	32	>64	16	64	1	2	11	C
Kp8	>128	>128	>128	>128	>128	>64	16	>64	64	>64	2	2	11	E
Kp9	>128	>128	>128	>128	>128	>64	32	>64	64	64	1	2	11	E
Kp10	>128	>128	>128	>128	>128	64	8	>64	32	64	0.5	2	37	A
Kp11	>128	>128	>128	>128	>128	>64	32	>64	64	64	0.25	2	11	E
Kp12	>128	>128	>128	>128	>128	>64	32	>64	64	64	>64	1	11	E
Kp13	>128	>128	>128	>128	>128	>64	32	>64	64	64	0.5	2	11	E
Kp14	>128	>128	>128	>128	>128	>64	32	>64	64	>64	0.25	2	11	E
Kp15*	>128	>128	>128	>128	>128	>64	16	>64	64	64	0.25	1	11	E
Kp16	64	4	>128	>128	>128	>64	32	64	4	4	0.125	2	37	A
Kp17	>128	>128	>128	>128	>128	>128	64	128	64	128	0.5	2	11	E
Kp18*	>128	>128	>128	>128	>128	>128	64	128	64	128	1	2	11	E

Kp19	>128	>128	>128	>128	>128	>64	64	64	64	64	0.125	2	11	D
Kp20	>128	>128	128	>128	>128	>64	64	>64	64	>64	0.25	2	11	D
Kp21	>128	>128	>128	>128	>128	>64	64	64	64	64	32	1	11	D
Kp22	2	>128	>128	>128	128	>64	128	>64	128	>64	<0.06	2	11	E
Kp23	0.5	>128	>128	>128	128	>64	128	>64	128	>64	0.25	8	11	E
Kp24	>128	>128	>128	>128	>128	>128	32	>128	64	128	1	1	11	D
Kp25	>128	>128	>128	>128	>128	>128	32	>128	64	128	0.25	1	11	D
Kp26	>128	>128	>128	>128	>128	>128	32	>128	128	128	0.25	1	11	D
Kp27	0.5	0.25	>128	>128	>128	>128	16	>128	128	16	1	2	11	E
Kp28	0.5	0.25	>128	>128	>128	64	16	128	64	32	1	2	11	E
Kp29	0.5	0.25	>128	>128	>128	>128	32	>128	128	32	1	2	11	E

GEN: Gentamicin, TOB: Tobramycin, AMP: Ampicillin, CEZ: Cefazolin, CAZ: Ceftazidime, CTRX: Ceftriaxone, IMP: Imipenem, ETP: Ertapenem, LVFX: Levofloxacin, CPFX: Ciprofloxacin, PLB: Polymyxin B, TGC: Tigecycline

Asterisk: Hypermucoviscous *Klebsiella pneumoniae*

1.4 Supplementary Figure S1.

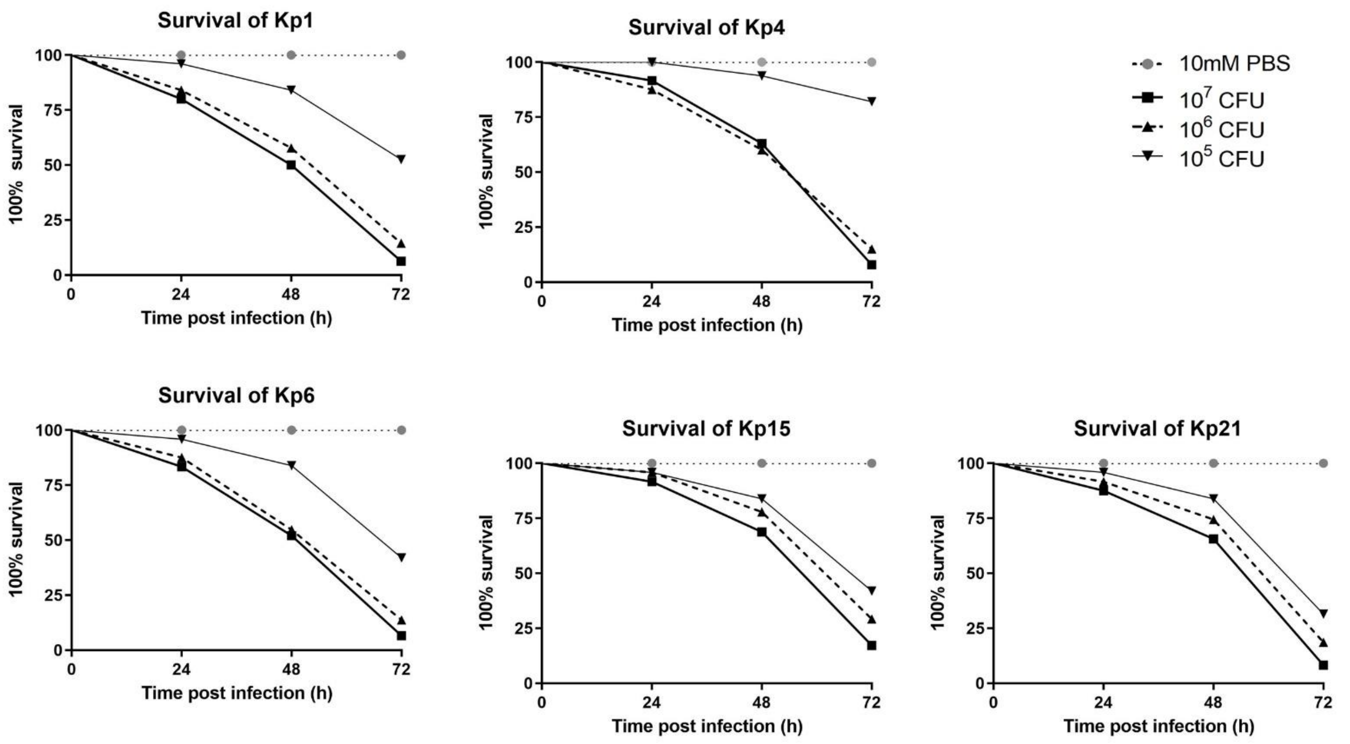

Supplement Figure S1. *K. pneumoniae* infection of *G. mellonella* larvae induces dose-dependent lethality. The effect of varies colony-forming units (CFU) of each *K pneumoniae* strain on survival was observed in *G mellonella*. Larvae were injected with PBS or with 10^5 /10^6 /10^7 CFU of Kp1, Kp4, Kp6, Kp15 and Kp21. The survival was monitored over 72 h post-infection. Mortality of larvae infected with Kp1, Kp4, Kp6, Kp15 and Kp21 were dose-dependent.
